# Severe Presentation and Delayed Intervention in Herpetic Gingivostomatitis

**DOI:** 10.7759/cureus.111757

**Published:** 2026-06-29

**Authors:** Maryam Sajjad, Hajer Alkazzaz, Esraa Sambas, Egab Al-Dowsari

**Affiliations:** 1 Pediatrics, Alfaisal University College of Medicine, Riyadh, SAU; 2 Medicine, King Saud University, Riyadh, SAU; 3 Pediatrics, Riyadh Second Health Cluster, Riyadh, SAU; 4 Pediatrics, King Fahad Medical City, Riyadh, SAU

**Keywords:** acyclovir, case report, hemorrhage, herpetic gingivostomatitis, hsv-1, oral lesions, pediatrics

## Abstract

Herpetic gingivostomatitis (HGS) is a common manifestation of herpes simplex virus type 1 (HSV-1) infection in children and is usually mild and self-limiting. We report the case of an eight-year-old boy with a known history of developmental delay who presented with prolonged fever and painful oral ulcers. The patient presented eight days after symptom onset. The lesions rapidly spread to involve the lips, tongue, and buccal mucosa, forming thick hemorrhagic crusts. He developed severe anemia that required transfusion and failed to improve with topical acyclovir and antibiotics. Upon presentation to our hospital, intravenous acyclovir was started with supportive therapy. His condition improved markedly, and the lesions resolved. This case describes an unusual, severe, hemorrhagic presentation of HGS in an immunocompetent child. It highlights that systemic antiviral therapy can lead to clinical improvement even when initiated later in the disease course and underscores the need for further studies to define optimal management for severe cases.

## Introduction

Herpetic gingivostomatitis (HGS) is a common manifestation of primary herpes simplex virus type 1 (HSV-1) infection that typically occurs in children between six months and five years of age; however, it can occur in adolescents and adults [1,2]. Transmission occurs through direct contact with infected oral secretions or lesions [2]. It presents with fever, cervical or submandibular lymphadenopathy, and painful clusters of vesicles and ulcers involving the oral mucosa, gingiva, palate, buccal surfaces, and the lips. These vesicles frequently rupture to form shallow ulcers [1,2].

Diagnosis is primarily clinical, relying on detailed patient history and physical examination, although laboratory tests may be performed to confirm when the diagnosis is unclear [2,3]. In severe presentations, the differential diagnosis may include reactive infectious mucocutaneous eruption (RIME), Stevens-Johnson syndrome, and other causes of oral mucosal ulceration [4,5]. These conditions may share similar clinical features with HGS and can make diagnosis more challenging.

In most cases, HGS is mild and self-limiting within 10-14 days [6,7]. Supportive care and maintenance of adequate hydration are the cornerstones of management, aided by effective pain control through oral analgesics and soothing mouth rinse [1,2]. Early recognition is important, as antiviral therapy may shorten symptom duration and improve clinical outcomes [3].

Hospital admission is considered for patients unable to maintain hydration as well as for those who are immunocompromised or who develop serious complications such as eczema herpeticum, encephalitis, or pneumonitis [2].

We report an unusual case of severe HGS presenting with extensive hemorrhagic oral lesions resulting in severe anemia that required packed red blood cell transfusion and had a more prolonged clinical course than typically observed in HGS. This case highlights the potential for significant systemic involvement even in immunocompetent children and underscores the importance of timely systemic antiviral treatment and supportive care.

## Case presentation

**Figure 1:**
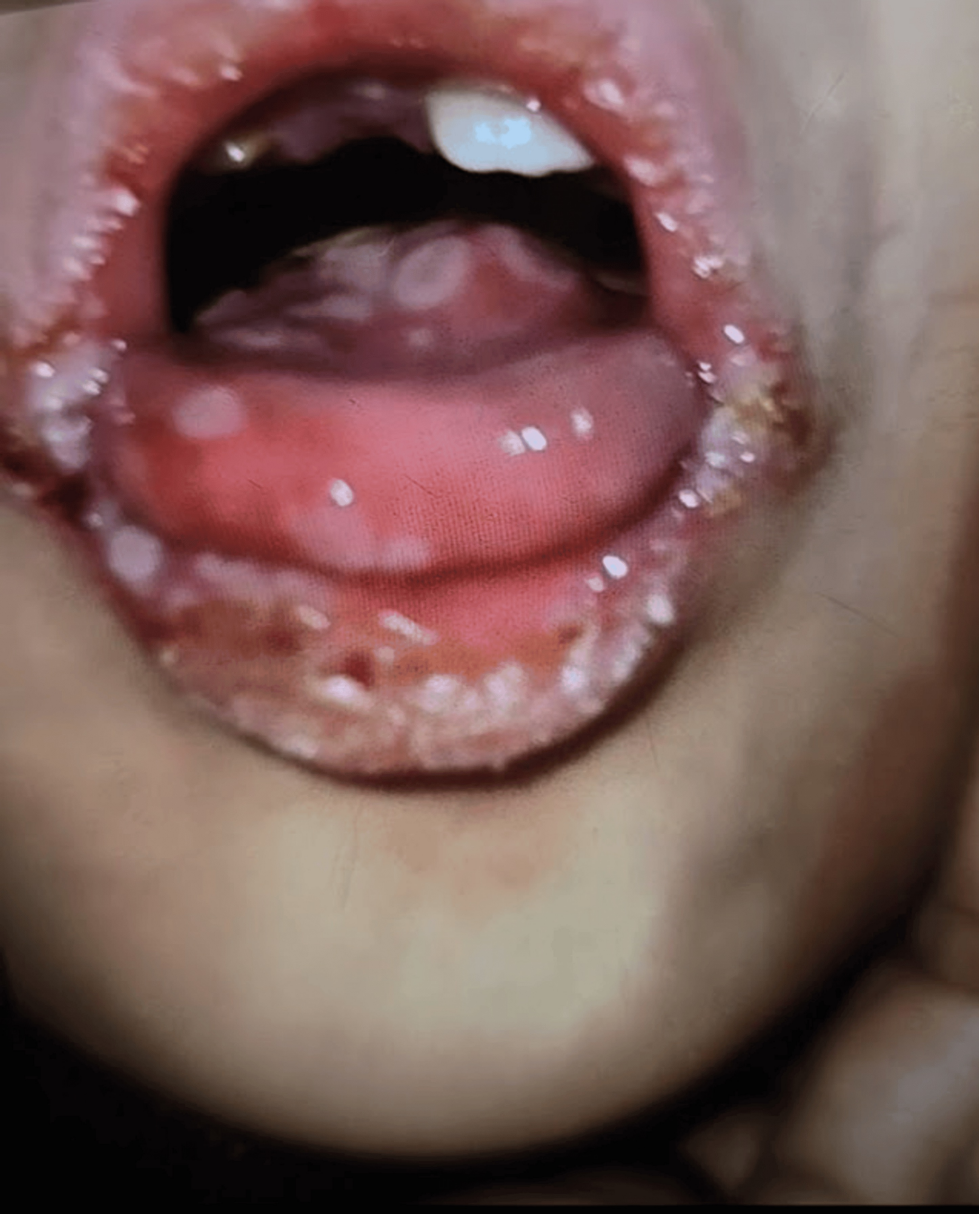
Vesicular and ulcerative oral lesions in primary herpetic gingivostomatitis Multiple white vesicles and ulcerative lesions of the lips. Numerous erosive plaques on the tongue, buccal mucosa, and hard palate.

**Figure 2:**
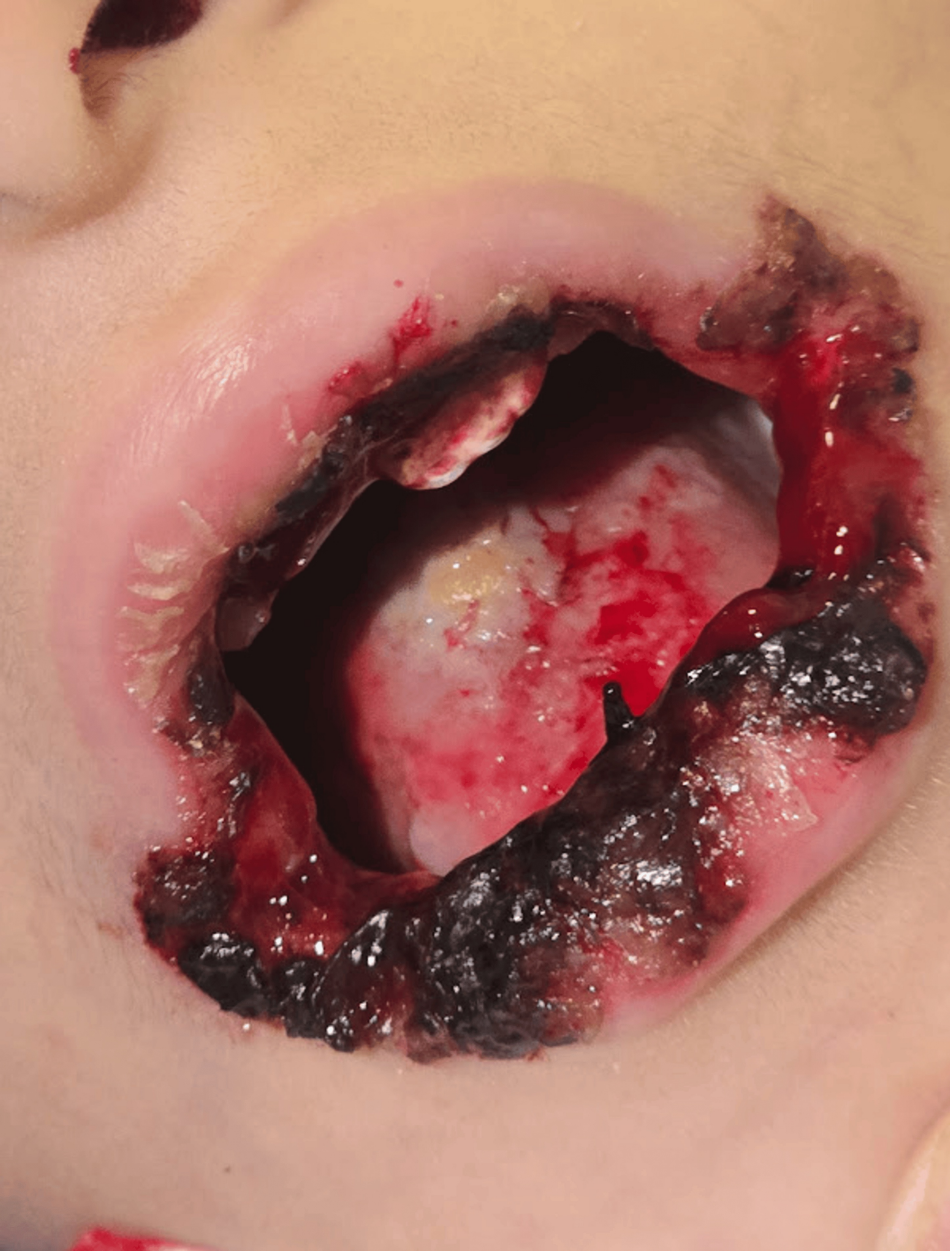
Ulcerative lesions of the lips with necrotic slough Hemorrhagic crusting of the lips. Upper and lower lip ulcerative lesions with thick yellow-brown crusts, necrotic slough, and surrounding mucosal erythema, crossing the vermilion border.

An eight-year-old boy with global developmental delay and bilateral cochlear implants presented with an 11-day history of painful oral ulcers and a nine-day history of oral thrush. He also had a fever during the first 10 days of illness, which had resolved before presentation. There were no reported respiratory symptoms, rashes, or sick contacts. Paracetamol and azithromycin were first given to him by his mother without a prescription. On the second day after initiating azithromycin, tiny white ulcers began to form on the lips and progressed to involve the tongue, buccal mucosa, and palate (Figure 1). They eventually developed into thick hemorrhagic crusts (Figure 2). He grew irritable, refused to eat, and lost approximately 2 kg within 14 days. He had spent six days in another hospital receiving intravenous (IV) fluids, topical acyclovir, and topical antifungals, and was administered IV amoxicillin-clavulanate for two days. Despite this, his fever and mouth lesions persisted, and his hemoglobin level dropped to 5 g/dL, necessitating a blood transfusion. Then the patient was admitted to our hospital.

Upon arrival at our emergency department, the patient was afebrile and hemodynamically stable. Examination, which was limited due to pain and irritability, revealed bloody saliva, extensive erosive oral ulcerations involving the lips and tongue, diffuse hemorrhagic crusting of the vermilion border, and white plaques involving the palate and buccal mucosa, as shown in Figure 2. Examination of the other systems was normal. Laboratory findings included an automated white blood cell count of 11.9 × 10³/µL, hemoglobin of 7.9 g/dL, hematocrit of 22%, mean corpuscular volume of 51.3 fL, C-reactive protein level of 55.9 mg/L, and potassium level of 3.0 mmol/L.

He was admitted and managed as a case of HGS. IV acyclovir 5 mg/kg every eight hours was initiated, hydration was maintained, and pain was controlled. Laboratory investigations showed that the viral polymerase chain reaction for varicella zoster and respiratory viruses was negative, whereas the HSV-1 polymerase chain reaction was positive. Urine analysis and culture results were unremarkable. Echocardiography was performed to exclude atypical Kawasaki disease (KD) and revealed normal coronary arteries. 

Dermatology consultation suggested a RIME related to herpes simplex virus infection or, less likely, a fixed drug eruption secondary to azithromycin. However, HSV-1 PCR was positive, and the overall clinical presentation was most consistent with severe HGS. The patient was treated with IV acyclovir, nystatin suspension, IV hydration, topical fusidic acid, petroleum jelly, and magic mouthwash (lidocaine, diphenhydramine, and antacids). 

The patient’s condition improved with the healing of oral lesions and improved oral intake. He was discharged in a stable condition, with oral acyclovir for two weeks, topical Vaseline, fusidic acid as needed, and a three-month course of ferrous sulfate for anemia with an outpatient follow-up. The family received counselling on medication adherence, hydration, and warning signs that required urgent medical attention.

The patient was followed up after discharge, and his condition completely returned to baseline. His oral mucosa had fully healed, he resumed normal oral intake, and no recurrence of lesions or systemic symptoms was reported. A summary of the clinical timeline, treatment, and outcome is provided in Table 1.

**Table 1:** Clinical timeline of illness, treatment, and outcome HSV, herpes simplex virus

Day of illness	Clinical course	Treatment	Outcome
Day 1	Fever develops	Paracetamol given by mother	Persistent fever
Day 2	Oral lesions begin to appear	Azithromycin initiated by mother	Progressive oral involvement
Days 3-11	Hospitalized at outside facility for worsening oral ulcers and poor oral intake; progressive oral ulceration and worsening oral intake; Hb decreased to 5 g/dL	IV fluids, topical acyclovir, antifungal therapy; packed red blood cell transfusion	Persistent fever and lesion progression; Hb rose to 8.36 g/dL
Days 9-11	Ongoing severe oral disease	IV amoxicillin-clavulanate	Only fever ended on day 10; no other significant clinical improvement
Day 12 (admission to our center)	Extensive hemorrhagic crusting; bloody saliva; active mucosal bleeding; erosive oral ulceration	HSV PCR sent; IV acyclovir and supportive care initiated	Clinical improvement noted within 24 hours (Figure 2)
Day 13-15	Progressive healing of oral lesions	HSV-1 positive; continued IV acyclovir and supportive care	Improved oral intake and reduced bleeding
Day 16	Marked improvement with decreased lesions and no active bleeding	Discharged on oral acyclovir	Eating and drinking adequately
Follow-up	Complete resolution of oral lesions	Completed oral acyclovir course	Returned to baseline

Written informed consent for publication was obtained from the patient's father.

## Discussion

Primary HGS is common in children. It presents with prodromal symptoms followed by multiple vesicles on the lips and oral mucosa. These vesicles ruptured, forming hemorrhagic crusts on the vermilion border and ulcers within the oral cavity. Gingiva may also swell and bleed. Lesions may involve the buccal mucosa, vermilion border, hard palate, tongue, or gingiva. Prodromal symptoms include fever, loss of appetite, drooling, and difficulty eating and drinking due to oral pain [3]. Our patient’s condition was unusually severe. In addition to typical prodromal symptoms, the patient had lost approximately 2 kg and developed severe microcytic anemia (hemoglobin 5 g/dL) during the two-week illness that required blood transfusion at a previous hospital. His weight loss may have been due to poor oral intake and inability to drink water, which caused vomiting that lasted for two weeks. The cause of anemia in this patient was likely multifactorial; bleeding from the severe hemorrhagic oral lesions may have contributed to the fall in hemoglobin; however, the marked microcytosis (mean corpuscular volume (MCV) 51.3 fL) suggests that anemia may have already been present before the onset of illness. Although the patient had global developmental delay, his father denied any history of feeding difficulties, swallowing impairment, or picky eating. Since baseline blood counts, iron studies, and hemoglobin electrophoresis were not available, it is difficult to determine the exact cause of his anemia. Nevertheless, this highlights the importance of monitoring blood counts and nutritional status in children with severe oral disease and poor oral intake.

On the eighth day, the patient presented with diffuse, thick brown-black hemorrhagic crusting at the lip fissure, active bleeding of the lips and tongue, oral thrush with white plaques throughout the oral cavity, and mucosal erythema of the posterior oropharynx extending beyond the vermilion border.

Characteristic clinical features can differentiate HSV-1 from other herpesvirus infections, including varicella-zoster virus, cytomegalovirus, and Epstein-Barr virus. The confirmatory test for primary herpetic gingivostomatitis (PHSG) is polymerase chain reaction; however, clinical findings are often sufficient to establish a diagnosis and initiate treatment [7]. Other differential diagnoses include RIME, Kawasaki, Stevens-Johnson syndrome, and herpetiform aphthae [2,4-5]. A multidisciplinary approach may be helpful in establishing a definitive diagnosis.

RIME was a differential because of extensive oral mucositis and severe mucosal involvement; however, it was considered less likely because RIME typically presents with severe mucositis after a respiratory infection and commonly presents with conjunctival and genitourinary involvement and sometimes with cutaneous lesions. Our patient had no cough, rhinorrhea, or other respiratory symptoms besides fever, and the lesions were limited to the oral cavity. In addition, HSV-1 PCR was positive, supporting severe primary HGS as the more likely diagnosis [8]. 

Although KD and primary HGS may both present with prolonged fever and oral mucosal involvement, KD typically also presents with bilateral nonexudative conjunctivitis, rash, extremity changes, and cervical lymphadenopathy. Since our patient only had prolonged fever and oral mucosal involvement, incomplete KD was considered, and echocardiography was done to rule out KD. Because our patient lacked other features of KD, and echocardiography demonstrated normal coronary arteries, KD was considered unlikely [9].

A fixed drug eruption secondary to azithromycin was also considered because oral lesions developed shortly after medication exposure. However, it presents as localized, well-demarcated plaques that occur at the same site following re-exposure to the medication. In contrast, our patient had diffuse oral ulceration and widespread mucosal involvement without a prior history of similar episodes, making this diagnosis less likely [10].

Stevens-Johnson syndrome was also considered because of the patient's fever and painful oral lesions following medication exposure. However, it presents as widespread mucocutaneous involvement, including cutaneous lesions, ocular involvement, and genital mucosal disease. These features were absent in our patient, making Stevens-Johnson syndrome less likely [5].

Overall, the diagnosis of severe primary HGS was favored because the patient had a positive HSV-1 result, characteristic oral lesions, and no evidence of significant involvement of the eyes, skin, or genital mucosa that would support an alternative diagnosis [6]. 

Many studies have reported that oral acyclovir reduces symptom duration when started within the first three to four days of symptom onset for primary gingivostomatitis [3,11]. Acyclovir inhibits viral DNA synthesis and prevents HSV replication. Topical acyclovir is not recommended for gingivostomatitis but is primarily used to treat herpes labialis. This may explain the lack of clinical improvement after topical acyclovir treatment in a previous hospital [12].

There is a lack of studies reporting the effectiveness of IV acyclovir and its late initiation for primary gingivostomatitis. Oral acyclovir is widely accepted as an effective treatment for gingivostomatitis [3,11]; however, evidence supporting IV acyclovir for PHSG remains limited. The patient had numerous erosive plaques on the tongue, buccal mucosa, and hard palate, with necrotic slough. The patient’s lesions and clinical symptoms did not improve after eight days of topical acyclovir. Because of severe oral pain, inability to tolerate oral intake, and vomiting with drinking water, IV acyclovir was initiated along with supportive care, including IV hydration, pain control, nystatin, and topical treatments. His fever subsided within one day, and he tolerated oral intake within three days. Although the patient improved after IV acyclovir was started, he also received several supportive treatments at the same time, making it difficult to know how much each intervention contributed to his recovery. More studies should be done to assess the independent relationship between IV acyclovir and primary herpetic gingivostomatitis.

Most established evidence for the use of acyclovir in HGS supports the early initiation of therapy, typically within 72 hours of symptom onset [3,11]. There is limited evidence regarding the effectiveness of acyclovir when started later in the course of illness. In our case, the patient presented on the eighth day of symptom onset; however, acyclovir was initiated, and a marked and rapid improvement in symptoms was observed following treatment. Even though improvement was seen after the late administration of IV acyclovir, this was a single case, and the patient also received several supportive treatments at the same time, making it difficult to determine the effect of any single intervention. Nevertheless, this case suggests that delayed IV acyclovir may still have a role in some severe cases of HGS and highlights the need for further studies.

## Conclusions

This case presents severe HGS with failure of topical acyclovir therapy and delayed initiation of systemic acyclovir. Clinical improvement was observed after initiation of IV acyclovir together with supportive care despite presentation later in the disease course. However, as multiple interventions were administered simultaneously, the individual effect of IV acyclovir cannot be determined.

The severe anemia observed in this patient appeared to be multifactorial, highlighting the importance of monitoring hematological parameters in children with severe oral disease. Early recognition of the key clinical features of herpes simplex virus infection is important to support timely and appropriate management. Further studies are needed to better understand the effectiveness and optimal timing of IV acyclovir in severe presentations of HGS.
